# Cardiovascular disease risk disparities between immigrants and native Koreans: a population-based study in Gwangju, Korea

**DOI:** 10.4178/epih.e2025067

**Published:** 2025-12-08

**Authors:** Jung-Ho Yang, Yerin Choi, Ran Lee, Seong Eun Kim, Kyung-Hwa Park, Seong-Woo Choi, BongKyu Sun, Kyunghak Kim, Sun-Seog Kweon

**Affiliations:** 1 Department of Preventive Medicine, Chonnam National University Medical School, Hwasun, Korea; 2 Gwangju Institute of Public Health and Equity, Gwangju, Korea; 3 Gwangju Center for Infectious Disease Control and Prevention, Gwangju, Korea; 4 Department of Infectious Diseases, Chonnam National University Medical School, Gwangju, Korea; 5 Department of Preventive Medicine, Chosun University Medical School, Gwangju, Korea; 6 Center for Global Diaspora Studies, Chonnam National University, Gwangju, Korea

**Keywords:** Immigrant, Cardiovascular disease, Health disparities, Korea

## Abstract

**OBJECTIVES:**

Korea is becoming a multiethnic society, with immigrants comprising nearly 5% of the population. Evidence on cardiovascular disease (CVD) risk among immigrants remains limited.

**METHODS:**

We conducted a population-based study of 582 immigrants in Gwangju and 2,328 age-matched and gender-matched native Koreans (2022-2023). Immigrant data were obtained from direct health assessments, while native Korean data were drawn from the Korea National Health and Nutrition Examination Survey. CVD risk was estimated using the Framingham risk score (FRS) and pooled cohort equations (PCE). Logistic regression was employed to compare the odds of elevated risk (10-year CVD risk ≥7.5%), adjusting for socio-demographic and behavioral factors.

**RESULTS:**

Immigrants had a higher prevalence of hypertension (37.3 vs. 16.1%), diabetes (11.5 vs. 5.6%), poor self-rated health (69.6 vs. 61.3%), and unmet medical needs (30.9 vs. 8.9%), as well as lower rates of health checkups and cancer screening (all p<0.001), compared to native Koreans. Elevated CVD risk was more frequent in immigrants (FRS, 31.4 vs. 20.8%; PCE, 33.6 vs. 22.8%). The adjusted odds ratios (95% confidence intervals) were 1.47 (1.14 to 1.88) for FRS and 1.49 (1.07 to 2.08) for PCE. Disparities were greatest among women, adults ≥40 years, uninsured people, low-income groups, and migrants from Central Asia, Russia, and Africa.

**CONCLUSIONS:**

Immigrants in Korea face substantially higher CVD risk than native Koreans, particularly within socioeconomically vulnerable subgroups. Targeted prevention and policies addressing structural barriers are urgently needed.

## GRAPHICAL ABSTRACT

[Fig f2-epih-47-e2025067]

## Key Message

Immigrants in Korea face unmet healthcare needs, such as limited screening access, low disease awareness, and undertreatment of dyslipidemia. Our study shows consistently higher cardiovascular risk among immigrants compared with native Koreans, especially among women, older adults, uninsured individuals, and Central Asian migrants. These findings underscore the epidemiological importance of structural and socioeconomic disadvantages in shaping immigrant health disparities and emphasize the need for culturally tailored interventions and inclusive health policies to achieve cardiovascular health equity.

## INTRODUCTION

Over the past two decades, Korea has undergone a rapid demographic transformation, marked by a substantial rise in its foreign-born population. The nation is gradually transitioning into a multiethnic society, with immigrants comprising nearly 5% of total residents. According to Statistics Korea, the number of foreign residents exceeded 2.46 million in 2023, accounting for more than 4.8% of the total population, up from 1.8% in 2010 [1].

However, while various public health policies have begun to consider migrant populations, there remains a notable paucity of epidemiologic studies that systematically compare the health status and risk factors of migrants with those of the native Korean population. Most existing Korean studies on migrant health have focused on specific conditions, such as infectious diseases [2-4] and mental disorders [5,6]. In addition, much of the research has been limited to certain subpopulations—such as migrant workers [7-10], marriage migrant women [11], Korean adolescents with migrant parents [12], and North Korean refugees [13]—rather than encompassing the broader migrant community as a heterogeneous and generalizable group. As a result, comprehensive, population-based studies that systematically examine health differentials and risk profiles for non-communicable diseases between migrants and native Koreans are still lacking [14,15].

In contrast, numerous studies in the United States and Europe have highlighted health advantages and disadvantages experienced by migrant populations relative to native-born residents. For instance, the well-documented “healthy immigrant effect” describes how recent immigrants often display better health indicators than native populations, likely due to selective migration processes [16]. Nevertheless, such initial advantages tend to attenuate over time because of accumulated psychosocial stress, socioeconomic disadvantages, and systemic barriers to healthcare access—a phenomenon often conceptualized as the “weathering effect” [17]. Furthermore, the “salmon bias” hypothesis posits that some severely ill migrants may return to their country of origin, potentially leading to underestimation of morbidity and mortality among migrants in the host country [18]. Emerging evidence from Korea [19], China [20], and several European nations [21-23] also suggests a “protective integration effect,” wherein stronger social integration, such as stable employment, language proficiency, community participation, and cultural competence, may buffer against health deterioration among migrants. Despite these theoretical frameworks, empirical findings on migrant health differentials vary considerably across settings. A recent meta-analysis reported that migrant populations from Africa, South Asia, the Caribbean, and Eastern Europe exhibited higher cardiovascular mortality than native-born populations, whereas migrants from East Asia and Latin America had lower mortality [24]. In the United States, cardiovascular disease (CVD) mortality was higher among United States-born Mexican Americans than among Mexican-born Mexican Americans and non-Hispanic whites [25]. Studies from Canada [26] and Israel [27] have shown that CVD risk and incidence differ by migrant origin, with some groups—such as African or Eastern European migrants—exhibiting distinct patterns in disease burden and healthcare utilization. These discrepancies emphasize that migration-associated cardiovascular risk is influenced by complex interactions among ethnicity, migration history, socioeconomic conditions, and access to preventive and therapeutic health services [24].

By contrast, only a handful of small-scale studies in Korea have directly compared CVD risk between migrant and native Korean populations, with inconsistent findings [8,10,28]. Given the rapid growth and increasing heterogeneity of Korea’s migrant population, understanding the CVD risk profile in this group is crucial not only for equity but also for the long-term sustainability of the national health system. Therefore, this population-based study aims to assess CVD risk among diverse migrant subgroups residing in a metropolitan Korean city and to identify and quantify any health disparities by comparing these groups with the general Korean population using representative health survey data. This evidence may facilitate the development of more inclusive and effective public health policies for immigrants in Korea’s diversifying society.

## MATERIALS AND METHODS

### Study participants

This study targeted foreign residents of Gwangju Metropolitan City, Korea, who participated in a community-based health assessment between 2022 and 2024. A total of 647 migrant individuals were initially enrolled. After excluding 34 participants younger than 18 years and 31 with missing data on key variables, the final analytic sample comprised 582 participants. Immigrant participants were recruited specifically for this study, with participation encouraged through promotional activities and collaboration with local immigrant support organizations.

The study population included diverse groups of foreign residents: “Koryoin” (ethnic Koreans from the former Soviet Union, primarily from Commonwealth of Independent States countries such as Russia, Uzbekistan, and Kazakhstan; n=165), migrant workers (n=312), and other immigrants (n=105), including marriage migrants, international students, refugees, and employment-based migrants (e.g., language teachers). Both documented (n=498) and undocumented immigrants (n=84) were eligible.

All participants underwent standardized health assessments, including anthropometric measurements (height, weight, and waist circumference), blood pressure measurements, laboratory testing (e.g., glycated hemoglobin [HbA1c], lipid profiles, and antibody tests for infectious diseases), chest radiography, urinalysis, and a structured questionnaire. The questionnaire collected data on socio-demographic characteristics; perceived health (very good, good, fair, poor, or very poor); health insurance coverage (insured vs. uninsured); use of general health checkups in the previous year; participation in cancer screening programs; self-reported history of chronic diseases (yes vs. no); unmet medical needs during the past year (yes, no, or not answered); and perceived usual stress (extreme, middle-high, middle-low, or low). Monthly income was assessed at the individual level for immigrants, as many lived alone or with non-family members, whereas for native Koreans, household income was collected and then divided by the number of household members to ensure comparability. For other key variables, we used the same measurement items as those in the Korean National Health and Nutrition Examination Survey (KNHANES). All questionnaires were translated into participants’ native languages and administered face-to-face by trained native-language interpreters. Additional details on data collection procedures have been reported elsewhere [4].

For the comparison group, native-born Korean adults were selected from the 2022-2023 KNHANES. We performed 1:4 individual matching by age and gender to select 2,328 native Korean participants as the reference group.

### Cardiovascular risk factors

Key cardiovascular risk factors were assessed using standardized clinical definitions. Blood pressure was measured in the seated position with a calibrated automatic sphygmomanometer after at least 5 minutes of rest. Hypertension was defined as systolic blood pressure (SBP) ≥140 mmHg, diastolic blood pressure ≥90 mmHg, or current use of antihypertensive medication. Diabetes was defined as an HbA1c level ≥6.5% or current use of glucose-lowering medication. Venous blood samples were collected after an overnight fast and analyzed at a central laboratory. Dyslipidemia was defined as total cholesterol ≥240 mg/dL, low-density lipoprotein cholesterol ≥160 mg/dL, high-density lipoprotein (HDL) cholesterol <40 mg/dL for men or <50 mg/dL for women, triglycerides ≥200 mg/dL, or current use of lipid-lowering medication. Smoking status was self-reported and categorized as current, former, or never smoker.

### Cardiovascular risk prediction models

The 10-year risk of CVD was estimated using 2 validated models: the Framingham risk score (FRS) [29] and pooled cohort equations (PCE) [30]. Both models included the following variables: age, total cholesterol, HDL cholesterol, SBP (treated or untreated), and smoking status; race was additionally included in the PCE. Each variable was scored according to the original algorithms, and the sums were converted to predicted 10-year CVD risk percentages. For analytic purposes, risk was considered a binary outcome, with elevated risk defined as an estimated 10-year CVD risk ≥7.5%. The thresholds were selected to ensure adequate outcome numbers and statistical power, corresponding to the upper tertile for FRS and the intermediate-risk criterion for PCE. The applicable age ranges differed across the models (FRS, 30-79 years; PCE, 40-79 years), which contributed to variations in the sample size for each model. Detailed formulas and risk criteria for both prediction models are available in the original publications [29,30], and the distribution of study participants by model is provided in the Supplementary Materials 1 and 2.

### Statistical analysis

Descriptive statistics were used to summarize participant characteristics. Categorical variables were compared between immigrants and native Koreans using chi-square tests or the Fisher exact test, while continuous variables were compared using independent t-tests. Logistic regression was used to evaluate the association between migration status and elevated CVD risk, adjusting for covariates that differed significantly between groups (perceived health, body mass index [BMI], health checkup in the preceding year, and unmet medical needs).

To examine whether the association varied within the migrant group, subgroup analyses were conducted using logistic regression, stratified by age group, gender, socioeconomic status, visa status, health behaviors, and region of origin. Adjusted odds ratios (aORs) and 95% confidence intervals (CIs) were calculated. In addition, the distribution of estimated 10-year CVD risk was assessed using kernel density estimation by gender and risk model. All analyses were conducted using R version 3.6.3 (R Foundation for Statistical Computing, Vienna, Austria), and statistical significance was set at a 2-sided p-value of less than 0.05.

### Ethics statement

The study protocol was approved by the Institutional Review Board of Chonnam National University Hospital (CNUH 2022-365), and written informed consent was obtained from all participants.

## RESULTS

Among the 582 immigrant participants, 56.0% were women, and over half (54.8%) were aged 19 years to 39 years. More than half (54.3%) had resided in Korea for at least 5 years, and 85.6% were documented migrants. A majority (55.3%) reported a monthly household income below 1.5 million Korean won. By region of birth, South Asians comprised the largest subgroup (48.5%), followed by Central Asians/Russians (25.4%) and East Asians (17.5%). Overall, 63.6% were covered by health insurance, while 36.4% were uninsured (Table 1).

Compared with native Koreans, immigrants were more likely to report poor self-rated health (69.6 vs. 61.3%) and to have a BMI ≥25.0 kg/m^2^ (39.5 vs. 34.1%), but less likely to have received a general health checkup within the past year (33.3 vs. 68.8%) or to have participated in cancer screening (13.2 vs. 53.0%). Unmet medical needs were reported more frequently among immigrants (30.9 vs. 8.9%). The prevalence rates of hypertension (37.3 vs. 16.1%) and diabetes mellitus (11.5 vs. 5.6%) were also significantly higher among immigrants (p<0.001 for all). Additionally, mean SBP (130.6±9.4 mmHg) and total cholesterol (196.8±39.1 mg/dL) were higher, and mean HDL cholesterol (54.0±13.1 mg/dL) was lower, in immigrants compared with native Koreans (p<0.05 for all; Table 2).

Across both CVD risk prediction models, the prevalence of elevated CVD risk (≥7.5% predicted 10-year risk) was consistently higher in immigrants than in native Koreans: 31.4% versus 20.8% for FRS and 33.6% versus 22.8% for PCE (p<0.001 for both; Table 3).

In multivariable logistic regression adjusted for covariates, immigrant status was significantly associated with elevated CVD risk in both models, with aOR (95% CI) values of 1.47 (1.14 to 1.88) for FRS and 1.49 (1.07 to 2.08) for PCE. Subgroup analyses showed that excess CVD risk among immigrants was more pronounced in women; older immigrants (≥40 years); those residing in Korea for more than 5 years; Koryoin; uninsured immigrants; lower income groups; and migrants from Central Asia, Russia, Africa, and other minority regions. These associations were generally consistent across both risk models (Table 4).

**Figure 1.**
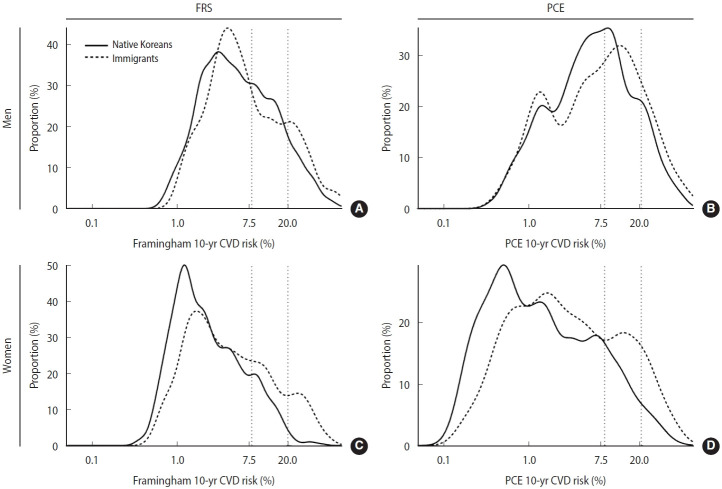
Gender-specific distributions of 10-year CVD risk according to the FRS (A: men, C: women) and PCE (B: men, D: women) models. CVD, cardiovascular disease; FRS, Framingham risk score; PCE, pooled cohort equations.

Regardless of gender, immigrants tended to have higher estimated CVD risk than native Koreans. In men, immigrants displayed a higher proportion in the FRS high-risk category and a right-shifted distribution on PCE. In women, across both models, immigrants consistently had higher proportions in the intermediate-risk and high-risk categories. Vertical dotted lines indicate cutoff values for intermediate risk (7.5%) and high-risk (20.0%) (Figure 1).

## DISCUSSION

In this population-based study of immigrants living in a metropolitan city in Korea, we found higher prevalence of hypertension, diabetes, and obesity, along with more adverse lipid profiles (higher total cholesterol, lower HDL cholesterol) than in native Koreans. Immigrants were also less likely to have received health checkups or cancer screenings and more likely to report unmet medical needs. Although the prevalence of dyslipidemia was slightly lower, this likely reflects markedly lower treatment rates (4.1 vs. 10.5%) rather than a truly lower burden; this difference was also statistically insignificant. Given the more atherogenic lipid patterns among immigrants, the reduced prevalence under the combined laboratory and treatment definition likely indicates underdiagnosis and undertreatment of lipid disorders. This may be driven in part by low awareness of dyslipidemia among immigrants due to limited access to screening, health information barriers, and insufficient culturally tailored education. Consequently, immigrants exhibited an elevated risk of CVD relative to their native counterparts. These disparities were especially pronounced among more vulnerable immigrants, such as women, older immigrants, uninsured individuals, those with lower incomes, and non-economic long-term settlement migrants such as Koryoin, whose primary purpose is long-term settlement rather than employment. These findings suggest that structurally and socioeconomically vulnerable subgroups bear a disproportionate CVD burden compared with both native Koreans and other immigrant groups.

Our findings align with international research showing that migrants often face increased CVD risk due to structural disadvantages and limited access to preventive healthcare [24,26]. Consistent with studies in Europe and North America [16,25], our results suggest that the “healthy immigrant effect” may not persist over time, particularly among women and older adults. Notably, the elevated CVD risk observed among Central Asian migrants and uninsured individuals mirrors earlier Korean findings in undocumented or socioeconomically marginalized groups [6,28]. In contrast, prior Korean studies that focused on specific migrant subpopulations, such as migrant workers or marriage migrants, have reported inconsistent associations between migration status and cardiometabolic burden [8,11,14]. A major strength of the present study is the inclusion of a diverse immigrant sample, including Koryoin, undocumented migrants, and long-term residents, which provides a more representative assessment of immigrant cardiovascular risk. By directly assessing multiple cardiovascular risk factors and including subgroups that better reflect real-world immigrant experiences, the study protocol reduced information bias and increased representativeness. These methodological strengths may explain why our findings were more consistent and robust than those of previous Korean studies that examined narrower subpopulations.

Multiple mechanisms may underlie these disparities. At the individual level, the higher prevalence and poorer control of hypertension and diabetes among immigrants [14,31] may reflect ethnic-specific genetic susceptibilities, lifestyle factors (e.g., dietary habits), exposure to high-risk environments before and after migration, and lower engagement in preventive health behaviors [15,26]. Inadequate disease control may also be driven by barriers such as restricted healthcare access, low health literacy, and poor disease awareness, which together impede effective disease management [26,32]. Although behavioral risks (smoking, alcohol consumption, physical inactivity) varied by migration status, these factors alone did not fully explain the observed differences [28]. Additionally, psychosocial stress related to migration, language barriers, and cultural dissonance likely contributes to poorer health perceptions and unhealthy behaviors [10]. While we observed no significant difference in self-reported stress prevalence between immigrants and native Koreans, this finding should be interpreted with caution. Differences in stress sensitivity and awareness, along with the limitations of self-reported measures, may have attenuated potential disparities. Thus, the absence of statistical significance does not preclude a role for psychological stress in shaping health differences. Future studies using more sophisticated and validated stress measures are warranted to better capture these differences. Previous Korean studies of migrant workers reported lower CVD risk in migrant women than in native Korean women [8] or a smaller risk gap in women than in men [15]; in contrast, our study found larger and more consistent disparities among immigrant women. This discrepancy may be explained by the higher proportion of non-labor immigrant women in our sample, such as marriage or family reunification migrants, who undergo less stringent health selection and tend to have longer residence, increasing exposure to adverse lifestyle factors and barriers to healthcare access. These vulnerabilities are particularly evident among Koryoin immigrants, who include a high proportion of women and older adults migrating for medical or social welfare support or family reunification. Collectively, these factors may attenuate the healthy immigrant effect and contribute to the greater CVD risk disparities observed among women and Koryoin.

From a broader perspective, emerging epidemiologic frameworks indicate that disparities in cardiometabolic health are shaped primarily by interacting social determinants and structural inequities [24]. Structural factors, including legal status, health insurance coverage, occupational insecurity, and partial exclusion from national health systems, often intersect to produce cumulative disadvantage [31]. Life-course epidemiology underscores that exposures before, during, and after migration accumulate over time, amplifying chronic disease risk [33]. Thus, the elevated cardiovascular risk observed in immigrants should be interpreted as the outcome of a complex interplay among multilevel determinants, biological susceptibility, and systemic inequities.

Although social integration is generally protective for migrant health [20], such benefits may be attenuated in Korea, where a fragmented health system and limited entitlement to care (particularly for undocumented or precariously employed migrants) may undermine integration-related health gains [19]. In contrast, evidence from countries with more migrant-inclusive social and health policies—such as comprehensive integration measures, universal health coverage, and culturally tailored public health programs—shows measurable improvements in migrant health outcomes [21,34]. These findings highlight that stronger policy environments can mitigate long-term health disparities among immigrants. Policy-level barriers in Korea, including limited access to public health programs, a shortage of culturally and linguistically appropriate services, and inconsistent policy frameworks, may therefore sustain or even widen disparities, placing Korea at a disadvantage compared with nations that have adopted inclusive integration policies that demonstrably improve migrant health.

This study has certain limitations. First, the cross-sectional design precludes causal inference about the association between migration status and CVD risk. Second, reliance on self-reported measures (e.g., perceived health and health behaviors) may introduce recall or social desirability bias. Additionally, differences in data collection methods between immigrants and native Koreans, particularly the use of interpreter-assisted surveys among immigrants, may have led to misclassification or underreporting. Third, despite matching on age and gender, residual confounding by unmeasured variables (e.g., diet, physical inactivity, alcohol consumption, psychological stress) cannot be excluded. Information on alcohol consumption and physical inactivity was not collected in the immigrant survey, primarily due to cultural and religious considerations. The absence of these key cardiovascular risk factors may have limited our ability to fully adjust for lifestyle-related confounding. Moreover, socioeconomic variables could not be comprehensively adjusted for, given differences in income measurement and the limited comparability of education and occupation across immigrants and native Koreans. To address potential concerns about geographic mismatch between immigrants and native Koreans, we conducted a sensitivity analysis restricted to participants from Gwangju and the neighboring Jeonnam region. The results were consistent with the main findings, suggesting that regional differences had minimal impact on the observed disparities (Supplementary Material 3).

Despite these limitations, this study provides novel, population-based evidence on CVD risk disparities among immigrants in Korea. By integrating clinical and self-reported data across diverse migrant subgroups, including those often excluded from research, the findings highlight the urgent need for targeted, multilevel interventions. At the individual level, culturally tailored education and improved access to preventive screening programs may address low health literacy and modifiable behavioral risks. At the structural level, expanding insurance coverage, ensuring equitable access to public health services, and strengthening language interpretation services are critical to overcoming systemic barriers. Comprehensive policies that address the social determinants of health will be essential to achieving long-term cardiovascular health equity within migrant populations.

## Figures and Tables

**Figure f2-epih-47-e2025067:**
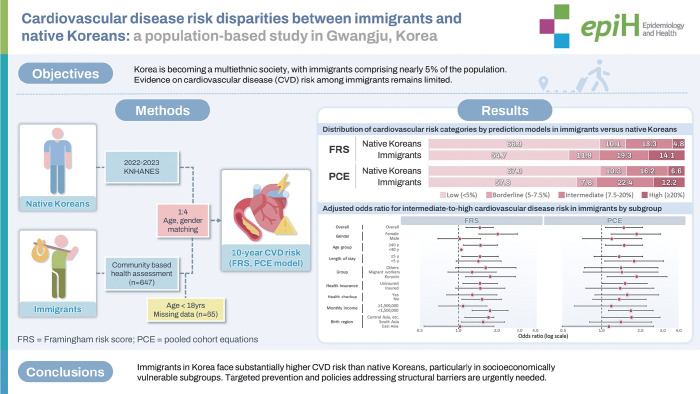


**Table 1.** Socioeconomic characteristics of immigrant participants in Gwangju, Korea (n=582)

Characteristics	Koryoin^1^	Migrant workers	Others^2^	Total
Total	165 (28.4)	312 (53.6)	105 (18.0)	582 (100)
Gender				
Men	51 (30.9)	174 (55.8)	31 (29.5)	256 (44.0)
Women	114 (69.1)	138 (44.2)	74 (70.5)	326 (56.0)
Age (yr)				
19-39	68 (41.2)	212 (67.9)	39 (37.1)	319 (54.8)
40-59	56 (33.9)	92 (29.5)	42 (40.0)	190 (32.6)
≥60	41 (24.8)	8 (2.6)	24 (22.9)	73 (12.5)
Length of stay (yr)				
<5	85 (51.5)	133 (42.6)	48 (45.7)	266 (45.7)
≥5	80 (48.5)	179 (57.4)	57 (54.3)	316 (54.3)
Immigration status				
Documented	152 (92.1)	250 (80.1)	96 (91.4)	498 (85.6)
Undocumented	13 (7.9)	62 (19.9)	9 (8.6)	84 (14.4)
Monthly income (KRW)				
<1,500,000	108 (65.5)	117 (37.5)	97 (92.4)	322 (55.3)
1,500,000-2,500,000	35 (21.2)	121 (38.8)	4 (3.8)	160 (27.5)
≥2,500,000	22 (13.3)	74 (23.7)	4 (3.8)	100 (17.2)
Country/region of birth				
East Asia^3^	0 (0)	77 (24.7)	25 (23.8)	102 (17.5)
South Asia^4^	0 (0)	216 (69.2)	66 (62.9)	282 (48.5)
Central Asia^5^, Russia	148 (89.7)	0 (0)	0 (0)	148 (25.4)
Africa	0 (0)	16 (5.1)	13 (12.4)	29 (5.0)
Others^6^	17 (10.3)	3 (1.0)	1 (1.0)	21 (3.6)
Health insurance status				
Insured	107 (64.8)	201 (64.4)	62 (59.0)	370 (63.6)
Uninsured	58 (35.2)	111 (35.6)	43 (41.0)	212 (36.4)

Values are presented as number (%). KRW, Korean won.

1 Koryoin: ethnic Koreans from the former Soviet Union mainly from Commonwealth of Independent States countries, e.g., Russia, Uzbekistan, Kazakhstan.

2 Others: marriage migrants, international students, refugees, and employment-based migrants (e.g., language teachers).

3 East Asia: China, Japan, and Mongolia.

4 South Asia: Vietnam, Laos, Malaysia, Indonesia, Cambodia, Thailand, Philippines, Nepal, Bangladesh, Sri Lanka, India, Myanmar, and Pakistan.

5 Central Asia: Uzbekistan, Kazakhstan, and Kyrgyzstan.

6 Others: North America, South America, Oceania, and Europe.

**Table 2.** Comparison of health indicators between immigrants and native Koreans

Variables	Immigrants (n=582)	Native Koreans (n=2,328)	p-value^1^	Variables	Immigrants (n=582)	Native Koreans (n=2,328)	p-value^1^
Age (yr)	40.8±13.9	40.8±13.9		Unmet dental needs			0.088
19-39	319 (54.8)	1,276 (54.8)		No	425 (73.0)	1,779 (76.4)	
40-59	190 (32.6)	760 (32.6)		Yes	157 (27.0)	549 (23.6)	
≥60	73 (12.5)	292 (12.5)		Hypertension			<0.001
Smoking status			0.335	No	365 (62.7)	1,953 (83.9)	
Current smoker	79 (13.6)	353 (15.2)		Yes	217 (37.3)	375 (16.1)	
Ex-/Never-smoker	503 (86.4)	1,974 (84.8)		Hypertension on medication			0.033
Perceived health			<0.001	No	509 (86.9)	2,095 (90.0)	
Good	177 (30.4)	900 (38.7)		Yes	76 (13.1)	233 (10.0)	
Poor	405 (69.6)	1,426 (61.3)		Diabetes			<0.001
Perceived usual stress			0.100	No	515 (88.5)	2,198 (94.4)	
No	419 (72.0)	1,594 (68.5)		Yes	67 (11.5)	130 (5.6)	
Yes	163 (28.0)	734 (31.5)		Diabetes on medication			0.003
BMI (kg/m^2^)			0.014	No	541 (93.0)	2,231 (95.8)	
<25	352 (60.5)	1,535 (65.9)		Yes	41 (7.0)	97 (4.2)	
≥25	230 (39.5)	793 (34.1)		Dyslipidemia			0.069
Health checkup			<0.001	No	483 (83.0)	1,852 (79.6)	
No	388 (66.7)	726 (31.2)		Yes	99 (17.0)	474 (20.4)	
Yes	194 (33.3)	1,602 (68.8)		Dyslipidemia on medication			<0.001
Cancer screening			<0.001	No	558 (95.9)	2,083 (89.5)	
No	505 (86.8)	1,094 (47.0)		Yes	24 (4.1)	245 (10.5)	
Yes	77 (13.2)	1,234 (53.0)		Systolic BP (mmHg)	130.6±19.4	115.7±14.2	<0.001
Unmet medical needs			<0.001	Total cholesterol (mg/dL)	196.8±39.1	192.6±37.1	0.019
No	402 (69.1)	2,120 (91.1)		HDL cholesterol (mg/dL)	54.0±13.1	59.4±16.1	<0.001
Yes	180 (30.9)	208 (8.9)					


Values are presented as mean±standard deviation or number (%). BMI, body mass index; BP, blood pressure; HDL, high-density lipoprotein.

1 Group differences were tested using the Pearson chi-square test or the Fisher exact test for categorical variables and the Welch 2-sample *t*-test for continuous variables.

**Table 3.** Distribution of cardiovascular risk categories by prediction model in immigrants compared to native Koreans

Variables	Immigrants	Native Koreans	p-value^1^
Framingham risk score (%)	446 (100)	1,784 (100)	<0.001
Low (<5.0)	244 (54.7)	1,193 (66.9)	
Borderline (5.0-7.5)	53 (11.9)	180 (10.1)	
Intermediate (7.5-20.0)	86 (19.3)	326 (18.3)	
High (≥20.0)	63 (14.1)	85 (4.8)	
Pooled cohort equations (%)	263 (100)	1,052 (100)	<0.001
Low (<5.0)	152 (57.8)	705 (67.0)	
Borderline (5.0-7.5)	20 (7.8)	108 (10.3)	
Intermediate (7.5-20.0)	59 (22.4)	170 (16.2)	
High (≥20.0)	32 (12.2)	69 (6.6)	

Values are presented as number (%).

1 Using the chi-square test.

**Table 4.** Cardiovascular disease risk in immigrants compared to native Koreans, overall and by subgroup^1^

Immigrant subgroup	FRS (n=446)	PCE (n=263)	Native Koreans (n=1,784/1,052)
Overall	1.47 (1.14, 1.88)	1.49 (1.07, 2.08)	1.00 (reference)
Gender			
Men	1.02 (0.69, 1.49)	1.20 (0.71, 2.05)	1.00 (reference)
Women	2.04 (1.44, 2.89)	1.86 (1.18, 2.90)	1.00 (reference)
Age (yr)			
<40	1.06 (0.36, 2.74)	N/A	1.00 (reference)
≥40	1.49 (1.09, 2.03)	1.49 (1.07, 2.08)	1.00 (reference)
Length of stay (yr)			
<5	1.44 (0.96, 2.16)	1.80 (1.06, 3.04)	1.00 (reference)
≥5	1.48 (1.06, 2.05)	1.35 (0.87, 2.08)	1.00 (reference)
Group			
Koryoin	1.77 (1.13, 2.79)	1.89 (1.10, 3.23)	1.00 (reference)
Migrant workers	1.29 (0.83, 1.98)	1.43 (0.72, 2.75)	1.00 (reference)
Others	1.63 (0.95, 2.81)	1.42 (0.73, 2.75)	1.00 (reference)
Health insurance status			
Insured	1.48 (0.95, 2.31)	1.39 (0.75, 2.56)	1.00 (reference)
Uninsured	1.48 (1.08, 2.02)	1.52 (1.01, 2.27)	1.00 (reference)
Health checkup			
No	1.52 (1.06, 2.18)	1.37 (0.85, 2.22)	1.00 (reference)
Yes	1.28 (0.81, 2.00)	1.58 (0.88, 2.79)	1.00 (reference)
Monthly income (KRW)			
<1,500,000	1.77 (1.28, 2.44)	1.69 (1.13, 2.52)	1.00 (reference)
≥1,500,000	1.09 (0.70, 1.67)	1.18 (0.61, 2.23)	1.00 (reference)
Country/region of birth			
East Asia	1.03 (0.54, 1.90)	1.14 (0.49, 2.55)	1.00 (reference)
South Asia	1.56 (1.05, 2.30)	1.41 (0.80, 2.47)	1.00 (reference)
Central Asia, etc.^2^	1.71 (1.12, 1.90)	1.71 (1.02, 2.86)	1.00 (reference)

Values are presented as adjusted odds ratio (95% confidence interval). FRS, Framingham risk score; PCE, pooled cohort equations; N/A, not applicable due to model age range restriction or insufficient event cases; KRW, Korean won.

1 Multivariate logistic regression adjusted for age, gender, perceived health, body mass index, perceived usual stress, health checkup and cancer screening in the last year, monthly income, and experience of unmet medical or dental needs; Subgroup-defining variables were excluded from the covariates in each model.

2 Central Asia, Russia, Africa, and others included.

## Supplementary materials

Supplementary Material 1. Comparison of health indicators between immigrants and native Koreans included in the FRS model

Supplementary Material 2. Comparison of health indicators between immigrants and native Koreans included in the PCE model

Supplementary Material 3. Sensitivity analysis of adjusted odds ratio for elevated cardiovascular disease risk in immigrants compared to native Koreans residing in Gwangju and Jeonnam, overall and by subgroup
