# Supplementary material for: Cardiovascular disease risk disparities between immigrants and native Koreans: a population-based study in Gwangju, Korea
Source: Epidemiol Health. 2025 Dec 8;47:e2025067. doi: 10.4178/epih.e2025067 (PMC12884040; doi:10.4178/epih.e2025067)
Supplement: Supplementary Material 1. — Comparison of health indicators between immigrants and native Koreans included in the FRS model [file epih-47-e2025067-Supplementary-1.docx]

**Supplementary Table S1. Comparison of health indicators between immigrants and native Koreans included in the FRS model**

|  | Immigrants  (n = 446) | Native Koreans  (n = 1,784) | p-value |
| --- | --- | --- | --- |
| Age (years) | 45.6 ± 12.4 | 45.6 ± 12.3 | 0.994 |
| Age group |  |  | >0.999 |
| 19-39y | 183 (41.0) | 732 (41.0) |  |
| 40-59y | 190 (42.6) | 760 (42.6) |  |
| ≥ 60y | 73 (16.4) | 292 (16.4) |  |
| Current smokers | 60 (13.5) | 239 (13.4) | 0.975 |
| Monthly income (KRW) |  |  | <0.001 |
| < 1,500,000 | 258 (57.8) | 573 (32.1) |  |
| 1,500,000-2,500,000 | 108 (24.2) | 556 (31.2) |  |
| ≥ 2,500,000 | 80 (17.9) | 655 (36.7) |  |
| Good perceived health | 134 (30.0) | 649 (36.4) | 0.012 |
| Having stress | 128 (28.7) | 542 (30.4) | 0.488 |
| BMI ≥ 25 kg/m^2^ | 198 (44.4) | 614 (34.4) | <0.001 |
| Received health checkup | 157 (35.2) | 1,310 (73.4) | <0.001 |
| Received cancer screening | 74 (16.6) | 1,134 (63.6) | <0.001 |
| Unmet medical needs | 137 (30.7) | 172 ( 9.6) | <0.001 |
| Unmet dental needs | 126 (28.3) | 447 (25.1) | 0.167 |
| Hypertension | 183 (41.0) | 357 (20.0) | <0.001 |
| Hypertension on medication | 75 (16.8) | 229 (12.8) | 0.028 |
| Diabetes | 62 (13.9) | 119 ( 6.7) | <0.001 |
| Diabetes on medication | 40 ( 9.0) | 94 ( 5.3) | 0.003 |
| Dyslipidemia | 82 (18.4) | 446 (25.0) | 0.003 |
| Dyslipidemia on medication | 24 ( 5.4) | 244 (13.7) | <0.001 |
| Systolic BP(mmHg) | 131.6 ± 20.5 | 116.2 ± 14.9 | <0.001 |
| Total Cholesterol (mg/dL) | 198.1 ± 39.2 | 195.0 ± 38.3 | 0.136 |
| HDL-cholesterol (mg/dL) | 54.1 ± 13.4 | 59.6 ± 16.6 | <0.001 |

Values are mean ± standard deviation or number (%). Group differences were tested using the Pearson’s chi-square test or Fisher’s exact test for categorical variables and Welch Two-Sample t-test for continuous variables; FRS, Framingham Risk Score; BMI, body mass index; BP, blood pressure; HDL, high density lipoprotein
