# Supplementary material for: Cardiovascular disease risk disparities between immigrants and native Koreans: a population-based study in Gwangju, Korea
Source: Epidemiol Health. 2025 Dec 8;47:e2025067. doi: 10.4178/epih.e2025067 (PMC12884040; doi:10.4178/epih.e2025067)
Supplement: Supplementary Material 2. — Comparison of health indicators between immigrants and native Koreans included in the PCE model [file epih-47-e2025067-Supplementary-2.docx]

**Supplementary Table S2. Comparison of health indicators between immigrants and native Koreans included in the PCE model**

|  | Immigrants  (n = 263) | Native Koreans  (n = 1,052) | p-value |
| --- | --- | --- | --- |
| Age (years) | 53.2 ± 10.5 | 53.2 ± 10.5 | >0.999 |
| Age group |  |  | >0.999 |
| 19-39y | 0 ( 0.0) | 0 ( 0.0) |  |
| 40-59y | 190 (72.2) | 760 (72.2) |  |
| ≥ 60y | 73 (27.8) | 292 (27.8) |  |
| Current smokers | 29 (11.0) | 119 (11.3) | 0.896 |
| Monthly income (KRW) |  |  | <0.001 |
| < 1,500,000 | 172 (65.4) | 377 (35.8) |  |
| 1,500,000-2,500,000 | 60 (22.8) | 325 (30.9) |  |
| ≥ 2,500,000 | 31 (11.8) | 350 (33.3) |  |
| Good perceived health | 81 (30.8) | 360 (34.2) | 0.293 |
| Having stress | 74 (28.1) | 277 (26.3) | 0.554 |
| BMI ≥ 25 kg/m^2^ | 128 (48.7) | 347 (33.0) | <0.001 |
| Received health checkup | 93 (35.4) | 821 (78.0) | <0.001 |
| Received cancer screening | 48 (18.3) | 780 (74.1) | <0.001 |
| Unmet medical needs | 77 (29.3) | 98 ( 9.3) | <0.001 |
| Unmet dental needs | 71 (27.0) | 254 (24.1) | 0.338 |
| Hypertension | 134 (51.0) | 301 (28.6) | <0.001 |
| Hypertension on medication | 70 (26.6) | 216 (20.5) | 0.032 |
| Diabetes | 54 (20.5) | 108 (10.3) | <0.001 |
| Diabetes on medication | 36 (13.7) | 88 ( 8.4) | 0.008 |
| Dyslipidemia | 66 (25.1) | 362 (34.4) | 0.004 |
| Dyslipidemia on medication | 22 ( 8.4) | 226 (21.5) | <0.001 |
| Systolic BP(mmHg) | 135.7 ± 22.1 | 118.4 ± 15.9 | <0.001 |
| Total Cholesterol (mg/dL) | 203.0 ± 40.4 | 195.1 ± 40.6 | 0.005 |
| HDL-cholesterol (mg/dL) | 53.6 ± 13.3 | 60.0 ± 16.7 | <0.001 |

Values are mean ± standard deviation or number (%). Group differences were tested using the Pearson’s chi-square test or Fisher’s exact test for categorical variables and Welch Two-Sample t-test for continuous variables; PCE, Pooled Cohort Equations; BMI, body mass index; BP, blood pressure; HDL, high density lipoprotein
