# Supplementary material for: Cardiovascular disease risk disparities between immigrants and native Koreans: a population-based study in Gwangju, Korea
Source: Epidemiol Health. 2025 Dec 8;47:e2025067. doi: 10.4178/epih.e2025067 (PMC12884040; doi:10.4178/epih.e2025067)
Supplement: Supplementary Material 3. — Sensitivity analysis of adjusted odds ratio for elevated cardiovascular disease risk in immigrants compared to native Koreans residing in Gwangju and Jeonnam, overall and by subgroup [file epih-47-e2025067-Supplementary-3.docx]

**Supplementary Table S3.** Sensitivity analysis of adjusted odds ratio for elevated cardiovascular disease risk in immigrants compared to native Koreans residing in Gwangju and Jeonnam, overall and by subgroup

| Immigrants subgroup | OR (95% CI) | | Koreans  (n=327) |
| --- | --- | --- | --- |
|  | FRS  (n=269) | PCE  (n=224) |  |
| Overall | 2.19 (1.35 - 3.59) | 1.84 (1.13 - 3.02) | Reference |
| Gender |  |  |  |
| Men | 1.07 (0.43 - 2.63) | 1.28 (0.59 - 2.81) | Reference |
| Women | 2.98 (1.65 - 5.45) | 1.98 (1.04 - 3.82) | Reference |
| Age group (year) |  |  |  |
| <40 years | NA | NA | Reference |
| ≥40 years | 2.21 (1.36 - 3.63) | 1.84 (1.13 - 3.02) | Reference |
| Length of stay |  |  |  |
| < 5years | 2.25 (0.96 - 5.32) | 2.87 (1.25 - 6.79) | Reference |
| ≥ 5years | 2.04 (1.10 - 3.81) | 1.41 (0.76 - 2.65) | Reference |
| Group |  |  |  |
| Koryoin | 2.58 (1.18 - 5.78) | 2.38 (1.10 - 5.32) | Reference |
| Migrant workers | 1.97 (0.81 - 4.98) | 2.22 (0.77 - 6.67) | Reference |
| Others | 3.77 (1.05 - 14.17) | 2.38 (1.10 - 5.32) | Reference |
| Health insurance |  |  |  |
| Insured | 2.26 (1.23 - 4.22) | 1.90 (1.03 - 3.55) | Reference |
| Uninsured | 2.70 (1.05 - 7.27) | 2.26 (0.84 - 6.39) | Reference |
| Health checkup |  |  |  |
| No | 2.68 (1.52 - 4.81) | 1.67 (0.96 - 2.90) | Reference |
| Yes | 1.74 (0.82 - 3.75) | 2.58 (1.08 - 6.48) | Reference |
| Monthly income (KRW) |  |  |  |
| <1,500,000 | 3.32 (1.87 - 6.00) | 2.25 (1.27 - 4.07) | Reference |
| ≥ 1,500,000 | 1.10 (0.42 - 2.89) | 1.53 (0.53 - 4.49) | Reference |
| Country of birth |  |  |  |
| East Asia | 1.73 (0.53 - 5.85) | 1.12 (0.30 - 4.07) | Reference |
| South Asia | 2.17 (0.88 - 5.47) | 1.81 (0.70 - 4.74) | Reference |
| Central Asia etc.^1)^ | 2.19 (1.03 - 4.72) | 2.25 (1.06 - 4.90) | Reference |

Multivariate logistic regression adjusted for age, gender, perceived health, BMI, perceived usual stress, health checkup and cancer screening in the last year, monthly income, and experience of unmet medical or dental needs; Subgroup-defining variables were excluded from the covariates in each model; FRS, Framingham risk score; PCE, Pooled cohort equation; NA, not applicable due to model age range restriction or insufficient event cases; 1) Central Asia, Russia, Africa, others included
